# Water availability drives aboveground biomass and bird richness in forest restoration plantings to achieve carbon and biodiversity cobenefits

**DOI:** 10.1002/ece3.5874

**Published:** 2019-11-27

**Authors:** Valerie Hagger, Kerrie Wilson, Jacqueline R. England, John M. Dwyer

**Affiliations:** ^1^ School of Biological Sciences The University of Queensland Brisbane Qld Australia; ^2^ Institute for Future Environments Queensland University of Technology Brisbane Qld Australia; ^3^ CSIRO Land and Water Clayton South Vic. Australia; ^4^ CSIRO Land and Water Brisbane Qld Australia

**Keywords:** biodiversity conservation, bird richness, carbon sequestration, environmental plantings, productivity, reforestation, subtropical forest

## Abstract

To combat global warming and biodiversity loss, we require effective forest restoration that encourages recovery of species diversity and ecosystem function to deliver essential ecosystem services, such as biomass accumulation. Further, understanding how and where to undertake restoration to achieve carbon sequestration and biodiversity conservation would provide an opportunity to finance ecosystem restoration under carbon markets. We surveyed 30 native mixed‐species plantings in subtropical forests and woodlands in Australia and used structural equation modeling to determine vegetation, soil, and climate variables most likely driving aboveground biomass accrual and bird richness and investigate the relationships between plant diversity, aboveground biomass accrual, and bird diversity. We focussed on woodland and forest‐dependent birds, and functional groups at risk of decline (insectivorous, understorey‐nesting, and small‐bodied birds). We found that mean moisture availability strongly limits aboveground biomass accrual and bird richness in restoration plantings, indicating potential synergies in choosing sites for carbon and biodiversity purposes. Counter to theory, woody plant richness was a poor direct predictor of aboveground biomass accrual, but was indirectly related via significant, positive effects of stand density. We also found no direct relationship between aboveground biomass accrual and bird richness, likely because of the strong effects of moisture availability on both variables. Instead, moisture availability and patch size strongly and positively influenced the richness of woodland and forest‐dependent birds. For understorey‐nesting birds, however, shrub cover and patch size predicted richness. Stand age or area of native vegetation surrounding the patch did not influence bird richness. Our results suggest that in subtropical biomes, planting larger patches to higher densities, ideally using a diversity of trees and shrubs (characteristics of ecological plantings) in more mesic locations will enhance the provision of carbon and biodiversity cobenefits. Further, ecological plantings will aid the rapid recovery of woodland and forest bird richness, with comparable aboveground biomass accrual to less diverse forestry plantations.

## Graphical abstract

We assessed native mixed‐species plantings in subtropical forests and woodlands in Australia to determine vegetation, soil, and climate variables most likely driving aboveground biomass (AGB) accrual and bird richness and investigate the relationships between plant diversity, AGB accrual, and bird diversity. Our results suggest that in subtropical biomes, planting larger patches to higher densities, ideally using a diversity of trees and shrubs, in locations with higher water availability will enhance the provision of carbon and biodiversity cobenefits.

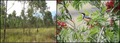

## 1 INTRODUCTION

Globally, there is an increasing need for landscape‐scale forest restoration to address land degradation, mitigate climate change, enhance food and water security, and combat biodiversity loss (IPCC, 2019; IPBES, 2018; Lamb, 2014; UNEP, 2019). Large‐scale reforestation is occurring worldwide, but many efforts involve planting productive monocultures (Chen et al., 2019; Hua et al., 2016) that may have limited benefits for biodiversity (Lindenmayer et al., 2012). In Australia, reforestation projects vary from native mixed‐species plantings that aim to reinstate habitat and enhance biodiversity (Hagger, Dwyer, & Wilson, 2017; Paul, Roxburgh, England, et al., 2015), to native forestry plantations and monocultures of eucalypts that aim to maximize timber yield or carbon sequestration (Paul, Roxburgh, de Ligt, et al., 2015; Stephens & Grist, 2014). Land managers in Australia can obtain payments for these planting types based on carbon sequestration predicted by the national carbon accounting model (DEE, 2014, 2017). Planting low‐diversity mixtures can reduce ecosystem stability and function, and the supply of ecosystem services, such as biomass accumulation, which plays a key role in carbon sequestration (Cardinale et al., 2012; Duffy, Godwin, & Cardinale, 2017). Therefore, understanding how and where to achieve both carbon sequestration and biodiversity enhancement could help fund ecosystem restoration projects through payments from carbon markets, where such markets exist.

Recent work has shown that native mixed‐species plantings have the potential to deliver biodiversity benefits alongside carbon abatement (Carwardine et al., 2015; Paul, Cunningham, et al., 2016; Pichancourt, Firn, Chades, & Martin, 2014). However, variation in the species planted, proportions of trees and shrubs (structural diversity), planting density, patch shape, and stand age can influence rates of aboveground biomass accumulation, as well as the quality of potential habitat for native fauna (Paul, Cunningham, et al., 2016; Paul, Roxburgh, England, et al., 2015). Aboveground biomass stores up to 50% carbon (Martin & Thomas, 2011) and is widely used as a measure of forest productivity and carbon stocks (Duffy et al., 2017; Lecina‐Diaz et al., 2018). Rates of aboveground biomass accumulation depend on climate and nutrient availability (Duffy et al., 2017; Ratcliffe et al., 2017; Vilà et al., 2013) and also vary among planting types. For example, in Australia, native mixed‐species plantings had comparable or lower aboveground biomass than eucalypt monocultures in temperate regions, but higher aboveground biomass in the tropics (Cunningham et al., 2015; Paul, Cunningham, et al., 2016). In subtropical China, aboveground carbon was positively related to planted species richness in a tree diversity experiment. After eight years, 16‐species mixtures had accumulated over twice the amount of carbon found in average monocultures and similar amounts to commercial monocultures (Huang et al., 2018). This suggests that more diverse forest plantings in subtropical Australia may also have greater rates of aboveground biomass accumulation.

Vegetation structure and diversity are often used as proxies for fauna habitat in studies of carbon and biodiversity cobenefits (Carwardine et al., 2015; Paul, Cunningham, et al., 2016; Pichancourt et al., 2014). However, for plantings to enhance biodiversity, they need to provide suitable habitat for, and be colonized by forest‐dependent fauna (Catterall, 2018). The composition and diversity of bird communities have been used to assess biodiversity recovery in natural regeneration and restoration plantings (Bowen, McAlpine, Seabrook, House, & Smith, 2009; Catterall, Freeman, Kanowski, & Freebody, 2012; Hale et al., 2015). Birds have been recognized as effective taxonomic surrogates for fauna diversity in agricultural landscapes and can achieve high representation of other taxa such as bees, reptiles, and arboreal marsupials (Ikin, Yong, & Lindenmayer, 2016; Li Yong et al., 2018).

Woodland and forest birds are declining worldwide, including in Australian eucalypt woodlands (Ford, 2011), and birds with particular life history traits are more at risk of decline in fragmented and degraded landscapes than other groups of bird species (Barnagaud, Barbaro, Papaïx, Deconchat, & Brockerhoff, 2014; Joyce, Barnes, Possingham, & Van Rensburg, 2018; Lindenmayer, Lane, et al., 2018). Understorey‐ and ground‐nesting birds are more susceptible to decline because of their dependence on understorey vegetation for nesting and shelter, and the widespread loss and degradation of the grass/tussock and shrub/sapling layers in agricultural landscapes that are subject to livestock grazing (Martin & McIntyre, 2007; Martin & Possingham, 2005; Shanahan, Possingham, & Martin, 2011). Loss and degradation of understorey vegetation has also been associated with declines in insectivorous birds because of reductions in insect diversity (Barton, Sato, Kay, Florance, & Lindenmayer, 2016; Gibb & Cunningham, 2010; White, Antos, Fitzsimons, & Palmer, 2005). Several studies also suggest that small‐bodied species are of conservation concern (Ford, Barrett, Saunders, & Recher, 2001; Montague‐Drake, Lindenmayer, & Cunningham, 2009), because of their poor ability to traverse fragmented landscapes compared to large‐bodied birds (Shanahan et al., 2011). Furthermore, where there is a lack of understorey vegetation in Australia, small‐bodied birds are more vulnerable to exclusion by aggressive competitors, such as the noisy miner (*Manorina melanocephala*; Maron et al., 2013), and ground‐nesters are particularly vulnerable to predation by feral cats (Woinarski et al., 2017).

Climate has been linked to patterns of bird diversity globally and in Australia (Coops, Rickbeil, Bolton, Andrew, & Brouwers, 2018; Hawkins, Diniz‐Filho, & Soeller, 2005; Hawkins, Porter, & Diniz‐Filho, 2003). It is thought that climate influences faunal diversity both directly (via the physiological requirements of animals which depend on ambient energy and water availability) and indirectly (through food availability, which depends on solar energy and water availability; Hawkins et al., 2003; Willig, Kaufman, & Stevens, 2003). In Australia, bird richness is strongly associated with evapotranspiration (a measure of water–energy balance), operating both directly and indirectly via plant productivity, as well as historical rainfall patterns (Coops et al., 2018; Hawkins et al., 2005).

Plant richness and vegetation structure are also known to influence bird diversity through provision of food and nesting sites, and protection from predators (Belder, Pierson, Ikin, & Lindenmayer, 2018; Bonifacio, Kinross, Gurr, & Nicol, 2011). Canopy cover and tree height have been found to be particularly important for woodland‐dependent birds, and the presence of a shrub layer influences colonization by understorey‐nesting birds (Barrett et al., 2008; Gould & Mackey, 2015; Munro et al., 2011). Older plantings support more bird species because more time has elapsed for species to colonize them, and because structural attributes for nesting and shelter have developed, such as large boughs and tree hollows (Kavanagh, Stanton, & Herring, 2007; Lindenmayer et al., 2010; Vesk, Nolan, Thomson, Dorrough, & Mac Nally, 2008; Whytock et al., 2018). However, disturbances such as from livestock grazing can negatively impact bird assemblages, particularly understorey‐dependent species, presumably through effects on vegetation structure (Martin & McIntyre, 2007). At broader spatial scales, larger restoration plantings and plantings with more surrounding native vegetation tend to have greater bird richness and abundance (Freeman, Catterall, & Freebody, 2015; Kavanagh et al., 2007; Lindenmayer, Blanchard, Crane, Michael, & Florance, 2018; Lindenmayer et al., 2010).

**Figure 1:**
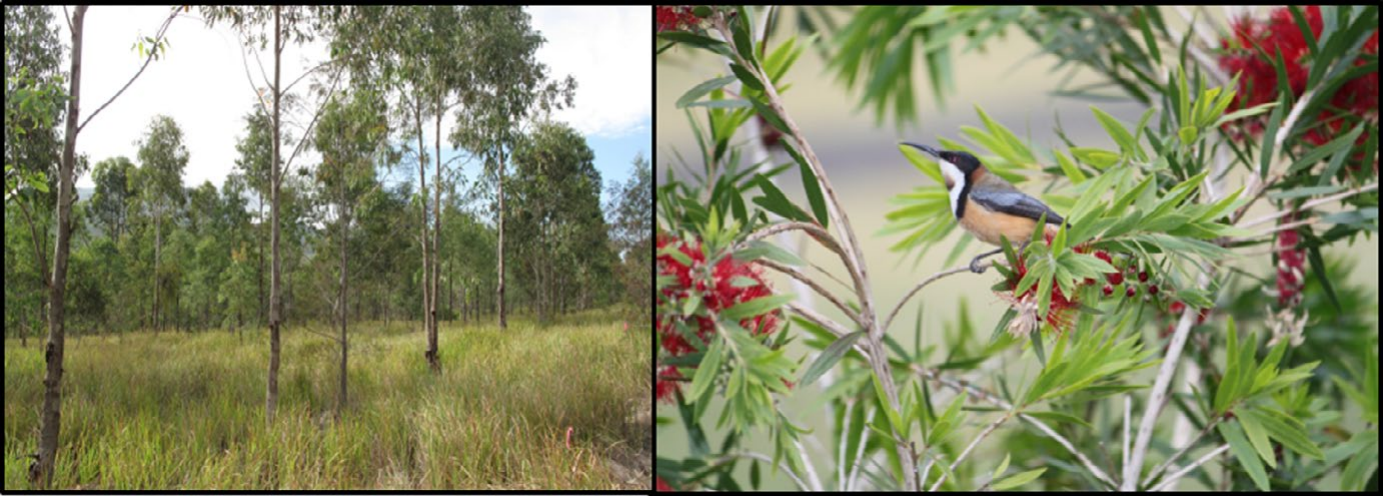
Subtropical forest planting in South East Queensland and resident forest‐dependent bird, Eastern Spinebill (*Acanthorhynchus tenuirostris*)

Despite the potential importance of plant diversity for enhancing productivity, and in turn bird diversity, the relationships in a restoration context are poorly understood. The objectives of our study were to identify the factors influencing both aboveground biomass production and bird diversity in subtropical forest plantings, and the synergies and trade‐offs between plant diversity, aboveground biomass production, and bird diversity. Realizing synergies will likely inform restoration projects (through site selection and planting design) to achieve carbon sequestration and biodiversity enhancement. We surveyed native mixed‐species plantings in subtropical Australia and used structural equation modeling (SEM) to determine the relationships between planting characteristics (species richness, stand density, and vegetation structure), aboveground biomass accrual (a proxy for productivity), and bird richness, and the influence of soil, climate, and landscape variables. We focussed on the species richness of woodland and forest‐dependent birds, and functional groups at risk of decline, including insectivorous, understorey‐nesting, and small‐bodied birds (example of forest planting and resident forest‐dependent bird in Figure 1). We hypothesized that plantings with higher species richness and structural complexity have greater aboveground biomass accrual and diversity of woodland and forest‐dependent birds, thus delivering carbon and biodiversity cobenefits.

## 2 MATERIALS AND METHODS

### 2.1 Study area

**Figure 2:**
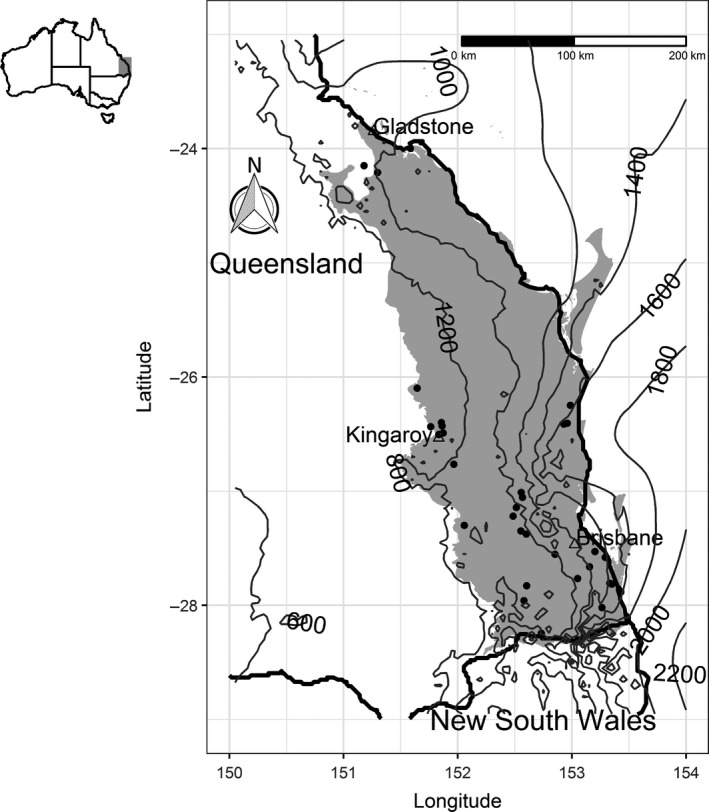
National and regional context of the restoration sites (black dots) surveyed in South East Queensland, Australia (gray area) showing mean annual precipitation isohyets (mm)

Our study area was the South East Queensland (SEQ) bioregion in Australia. The bioregion spans 62,484 km^2^ along the coast and adjacent hills and ranges, from the MacPherson Range and Border Ranges on the New South Wales border in the south, to Gladstone in the north. The Great Dividing Range extends north–south creating an altitudinal gradient from the coast (Figure 2). SEQ has a diversity of vegetation communities, including eucalypt forests and woodlands on lowlands, hills and ranges, wet forests on ranges and watercourses, melaleuca and mangrove forests, and coastal heathlands (Neldner et al., 2017). The bioregion has a humid subtropical climate, with mean annual precipitation (MAP) of 1,600 mm near the coast to 800 mm west of the Great Dividing Range (Figure 2), and mean annual temperatures ranging from 15–21°C (Bureau of Meteorology, 2019).

### 2.2 Restoration sites

We selected 30 native mixed‐species plantings located across SEQ in rural or peri‐urban areas that were established four or more years before the time of sampling, and greater than one hectare in size (Figure 2). Fourteen of these sites were part of an existing Australian research program designed to improve estimates of biomass accumulation (Paul et al., 2013). The remaining 16 sites were selected from discussions with local councils, state government, and private organizations. Plantings were established as low‐diversity forestry plantations (eucalypts only), mid‐diversity catchment improvement plantings (mixture of native trees), or species‐diverse ecological plantings (mixture of native trees and shrubs). Vegetation types were a mixture of wet forest, and eucalypt forest or woodland. Some sites were fenced to exclude livestock and macropods, while others were subject to periodic cattle grazing. Some of the younger sites were still under weed control at the time of sampling. Sites ranged from 4 to 25 years since planting, 1.4–158.8 ha in size, and MAP of 735–1,660 mm. Planting configurations were in blocks or patches (≥40 m in width as defined in Paul, Roxburgh, England, et al., 2015). Stand age was calculated from the year since planting to the year sampled.

### 2.3 Vegetation data

We surveyed vegetation structural and floristic attributes at all restoration sites following the BioCondition methodology (Eyre et al., 2015). At each site, a 100 m × 50 m plot was laid out, consisting of a 100 m transect perpendicular to tree rows or contours to capture variation in the species planted and 25 m either side of the transect. Recorded vegetation attributes were number of large trees with a diameter at breast height (DBH) greater than 30 cm, median canopy height of the ecologically dominant layer measured with a laser range finder (Nikon Corporation), and native and exotic tree species richness. Tree canopy cover and shrub canopy cover were measured as the vertical projection in meters over the 100 transect (proportion). Canopy and subcanopy layers were summed to give a total tree cover, and native and exotic shrubs were summed to give a total shrub cover. Native and exotic shrub species richness was recorded within a 50 m × 10 m subplot (incorporating 25–75 m along the transect, and encompassing 5 m either side of the transect).

Tree and shrub species richness and canopy cover were selected as measures of planting diversity and structural complexity. Pearson correlation coefficients (*r*) showed stand age to be highly correlated with tree height and number of large trees (*r* = .69 and .64, respectively); therefore, only stand age was selected as a surrogate for development of habitat.

We also recorded whether the sites had evidence of soil preparation (i.e., mounded or furrowed) or were currently managed for weeds, irrigated by effluent water, or allowed to have cattle grazing (binary variables). However, as cattle grazing was dominant in older sites, and weed control in younger sites, and there were few sites that had soil preparation or irrigation; these variables were not included in the analysis.

### 2.4 Biomass estimates

At each restoration site, we established two 50 m × 20 m plots, one inside the BioCondition plot, and the other to capture additional variation in trees planted. With sites that had very dense vegetation, plots were scaled down to 50 m × 10 m, and two sites were only large enough to have one plot. Within each plot, we identified and measured the diameter of all woody plants (trees and shrubs) greater than 1 cm, including dead stems. Tree diameter was measured at 130 cm height above ground level. For shrub and multistemmed species, diameter was typically measured at 10 cm height above the ground (Paul, Roxburgh, et al., 2016). For multistemmed individuals, a single diameter estimate was obtained from the quadratic mean (Chojnacky & Milton, 2008). Stand density was calculated as the mean number of trees and shrubs per hectare at each site (Paul, Cunningham, et al., 2016).

Stems were assigned one of five allometric equations to estimate their aboveground biomass, based on plant functional types (shrubs, multistemmed trees, single‐stemmed trees of the genus *Eucalyptus* and closely related genera, other trees of high wood density, and other trees of low wood density) developed to predict aboveground biomass for a range of ecoregions across Australia (Paul, Roxburgh, et al., 2016). The resulting stem biomass values (including dead stems) were summed across both plots and expressed as Mg ha^−1^ as an estimate of planting‐scale aboveground biomass. Planting‐scale aboveground biomass was divided by planting age to give annual aboveground biomass accrual (Mg ha^−1^ year^−1^), a proxy for productivity.

### 2.5 Bird data

We conducted three repeat bird surveys at all restoration sites during summer/early autumn (December 2017 to April 2018) within four hours of sunrise. Sites were surveyed by one observer with extensive ornithological field experience across SEQ and accompanied by a volunteer, walking variable paths throughout the patch, and the abundance of all bird species seen or heard recorded during a 30‐min period (Archibald, McKinney, Mustin, Shanahan, & Possingham, 2017; Martin & McIntyre, 2007). Given sites varied in size in fragmented landscapes, we used fixed time, rather than fixed area to standardize the scale of sampling. This fixed effort may have yielded samples of differing completeness, with samples from smaller, less complex sites being more representative, than samples from larger, more complex sites (Watson, 2003). Detectability was generally constant across the sites, because, although older sites had a higher canopy, they lacked a dense understorey, and birds were as visible as in younger, denser sites with a lower canopy height.

Estimates of abundance were based on the maximum number of birds seen at any one time to avoid double counting. Water birds and birds overflying were not included in the dataset, except for species that capture or search for their prey from the air (e.g., raptors and swallows). We avoided conducting surveys in heavy rain and strong wind. Animal ethics approval and scientific purposes permits were obtained prior to beginning the surveys.

We assigned bird species into habitat classifications devised by Fraser, Hauser, Rumpff, Garrard, and McCarthy (2017) for Australian terrestrial bird species. Classifications were specific to Australia and based on species occurrence data, percentage tree and woodland cover, and habitat preference across the whole of Australia, as well as three different ecoregions to account for regional differences in bird species' relationships with habitat. Bird species classified as “closed woodland,” “open woodland,” or “forest” were grouped as woodland and forest‐dependent. As our restoration sites were characterized by subtropical eucalypt forests and woodlands, and wet forests, we firstly used the classifications for the “temperate broadleaf and mixed forests” ecoregion. If a bird species was missing from this ecoregion, we used the classification for the “tropical and subtropical grasslands, savannahs, and shrublands” ecoregion, and then whole of Australia. If a bird species was missing from the dataset, we assigned the bird to a classification based on nesting, foraging and dispersal characteristics described in Marchant and Higgins (1990). Birds inhabiting wetlands and water environments were assigned “waterbird.”

We also assigned relevant bird species into the functional group classifications devised by Joyce et al. (2018) based on a trait analysis of bird species in Greater Brisbane, Australia: (a) “insectivores” that forage primarily on invertebrates and rarely on seeds, fruits, or other substrates; (b) “understorey‐nesting” that nest at or below 1.5 m, but not on the ground; (c) “ground‐nesting” that nest on or below the ground (in burrows); and (d) “small‐bodied” which weigh less than 67 g (median body size of the complete avian assemblage in the Greater Brisbane area). If a bird species was missing from the dataset, we assigned the bird to a functional group given descriptions in Marchant and Higgins (1990).

Based on the classifications above, we calculated multiple bird species richness variables for analysis. These included the richness of woodland and forest‐dependent species, insectivorous species, understorey‐nesting species, and small‐bodied species. We excluded ground‐nesting species richness, which included too many zeros to reliably analyze.

### 2.6 Soil data

Soil nutrients are considered essential for plant growth, and nitrogen (N) and phosphorus (P) can be limiting in Australian ecosystems (Cheesman, Preece, van Oosterzee, Erskine, & Cernusak, 2018; Crous, Ósvaldsson, & Ellsworth, 2015). Surface soil samples (0–10 cm depth) were collected every 10 m along each transect of the BioCondition plot using a handheld soil corer, and bulked into one sample to capture soil variation across the site (McKenzie, Henderson, & McDonald, 2002). Plant litter on the soil surface was scraped away before sampling. Samples were stored in plastic bags in an ice box in the field, and transferred to a cool, dry place until laboratory analysis. Samples were analyzed for Total N (%) and Organic Carbon (OC; %) using the Dumas combustion method, and extractable P (mg/kg) using the Colwell P method. Samples were prepared by oven drying (at least 48 hr at 40°C) and grinding to <2.0 mm for analysis of extractable P, and grinding to <0.5 mm for analysis of total N and OC. Total N and OC were highly correlated (*r* = .88), therefore Total N and P were selected to represent nutrient availability.

### 2.7 Climate data

Moisture availability is positively associated with bird richness across Australia, and provides a measure of ambient energy and water availability in the environment (Coops et al., 2018; Hawkins et al., 2005). Interpolated online climate data from SILO (Scientific Information for Landowners; DES, 2019; Jeffrey, Carter, Moodie, & Beswick, 2001) were used to calculate an index of moisture availability for each site. Moisture index was the ratio of mean annual precipitation to mean annual potential evapotranspiration for the growing period (year since planting to year sampled), calculated using Morton's potential evapotranspiration variable (Morton, 1983).

### 2.8 Landscape attributes

The size of the planting area, and the area in hectares of native vegetation within a 1 km radius of the center of the BioCondition plot were assessed in ArcMap 10.6 (ESRI, 2018a). World imagery basemap (ESRI, 2018b) was used to delineate the planting areas. Remnant Regional Ecosystem mapping (DES, 2018) and mature regrowth mapping (DES, 2013) were used to estimate the surrounding native vegetation.

### 2.9 Statistical analysis

All statistical analyses were undertaken in R 3.6.1 (R Core Team, 2019). First, we analyzed whether aboveground biomass accrual or bird richness varies significantly by planting type using an ANOVA. Pairwise differences between planting types were tested using the glht function in the multcomp package (Hothorn et al., 2016).

**Figure 3:**
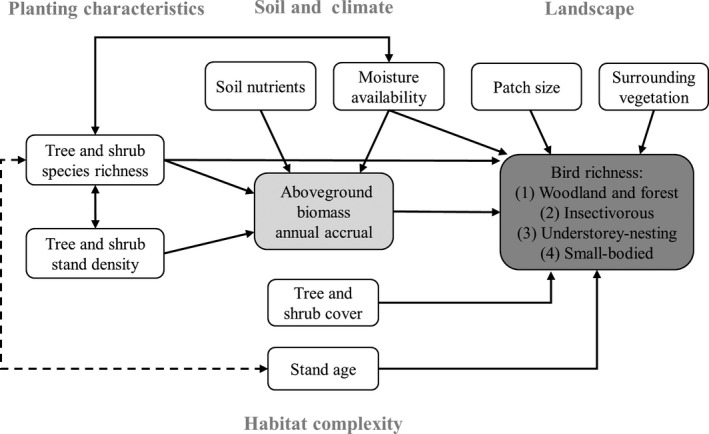
Structural equation meta‐model showing hypothesized relationships between variables. Single‐headed arrows indicate the direction of the causal relationship, and double‐headed arrows indicate the relationship is correlated. Black and dashed lines represent expected positive and negative effects, respectively

We used structural equation modeling (SEM) to test the direct and indirect relationships between aboveground biomass accrual, bird richness variables, selected vegetation attributes, and soil, climate, and landscape variables. We developed a causal graph, based on a priori expectations from a review of the literature, to describe the direction of hypothesized relationships between variables (Figure 3; Shipley, 2016). In this graph, bird richness variables were always treated as response variables. Aboveground biomass accrual was treated both as a response and explanatory variable. Remaining variables were treated as explanatory variables (in at least one component model), and included tree and shrub richness, stand density, shrub cover, soil nutrients (total N and extractable P), moisture index, patch size, amount of surrounding native vegetation, and stand age. Because most of the low‐ to mid‐diversity forestry or catchment plantings were older plantings, and most of the ecological plantings were located in coastal, wetter regions, there was a negative correlation between species richness and stand age, and a positive correlation between species richness and moisture index (*r* = .64), which we accounted for in the SEM.

**Figure 4:**
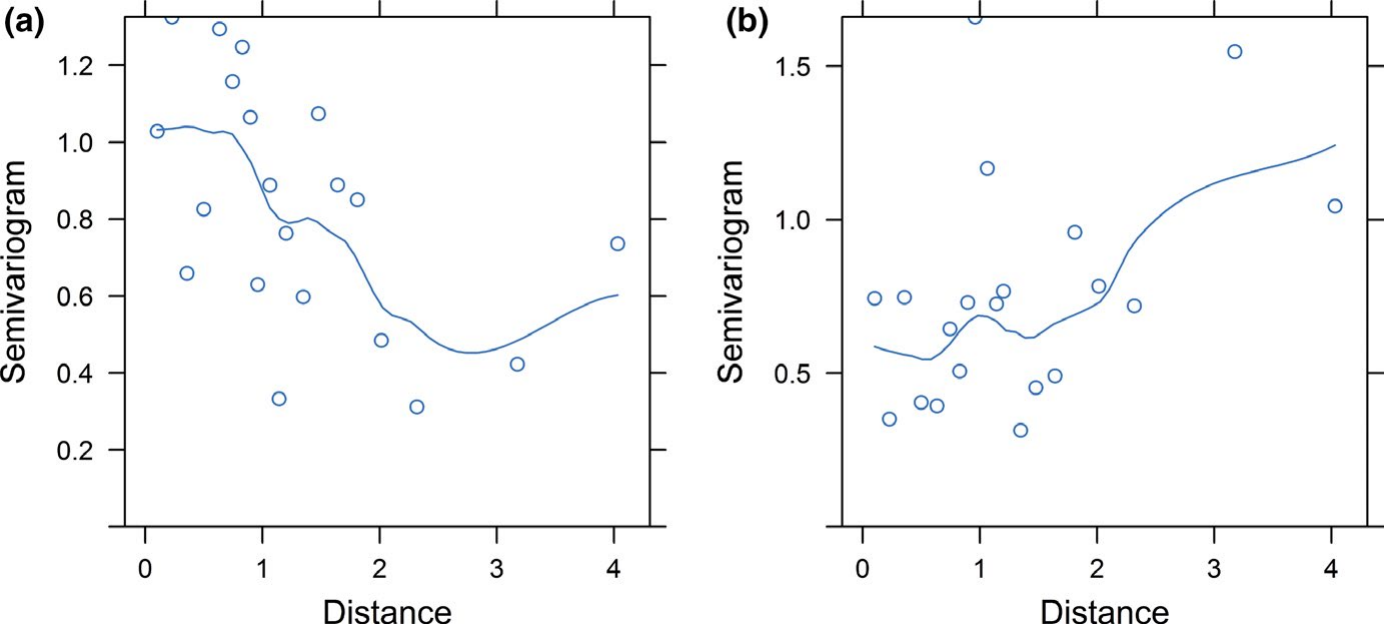
Variogram of a generalized least squares models of (a) aboveground biomass accrual component model and (b) woodland and forest bird richness component model with Gaussian errors, plotted with the site latitude and longitude, showing spatial autocorrelation among the restoration sites

Grace, Scheiner, and Schoolmaster (2015) recommend that the number of samples per parameter (*d*) does not fall below 5. In our SEM we had 11 parameters for a *d* of 2.73. Although *d* is marginal, we minimized over‐parameterization by focussing on a priori expectations and selecting independent explanatory variables. We used the “piecewiseSEM” package (Lefcheck, 2018) to test causal paths in our final SEM. We fitted four SEMs, one for each bird richness response variable (Table 1). Before fitting SEMs, component models for each response were inspected. Aboveground biomass accrual, and woodland and forest bird richness had normal distributions, therefore linear models were used for these component models. Residual spatial autocorrelation was present in aboveground biomass accrual, and woodland and forest bird richness among the sites (Figure 4), therefore we fitted spatial correlation structures to these component models in the woodland and forest bird richness SEM using generalized least squares (gls) models in the nlme package in R (Pinheiro, Bates, DebRoy, Sarkar, & Heisterkamp, 2019). However, the AIC_c_ weights showed no improvement in model fit, so we continued with the linear component models. Generalized linear models (GLMs) with Poisson errors and log link function were used for insectivore and understorey‐nesting bird richness, and GLM with quasi‐Poisson error was used for small‐bodied bird richness to account for overdispersion. Some explanatory variables were log transformed (stand density, moisture index, P, patch size) or square root transformed (surrounding native vegetation) to improve model fit and reduce the influence of larger values. Model coefficients were standardized to allow comparison across multiple responses in the causal network, and partial residuals were computed to facilitate plotting of significant relationships. This was not possible for understorey‐nesting, insectivore and small‐bodied bird richness because of transformation via the link function, and corresponding plots of significant relationships use the raw data. We evaluated SEMs using directed separation (d‐sep) tests based on Fisher's *C* statistic. Small *C* values, and consequently large *p*‐values (*p* > .05), indicate that the model fits the data well, and that independence claims (or causal paths not included in the model) are not significant.

**Table 1:** Response and predictor variables included in the component models for each structural equation model (SEM)

SEM	Response	Predictors
All four	Aboveground biomass accrual (Mg ha^−1^ year^−1^)	soil N (proportion) + log(soil P [mg kg^−1^]) + log(moisture index) + tree and shrub species richness + log(tree and shrub stand density [stems ha^−1^])
Woodland and forest bird SEM	Woodland and forest bird richness	Aboveground biomass accrual (Mg ha^−1^ year^−1^) + tree cover (proportion) + shrub cover (proportion) + tree and shrub species richness + log(patch size [ha]) + sqrt(native vegetation in 1 km buffer [ha]) + log(moisture index) + stand age (year)
Insectivorous bird SEM	Insectivorous bird richness	
Understorey‐nesting bird SEM	Understorey‐nesting bird richness	
Small‐bodied bird SEM	Small‐bodied bird richness	

## 3 RESULTS

**Figure 5:**
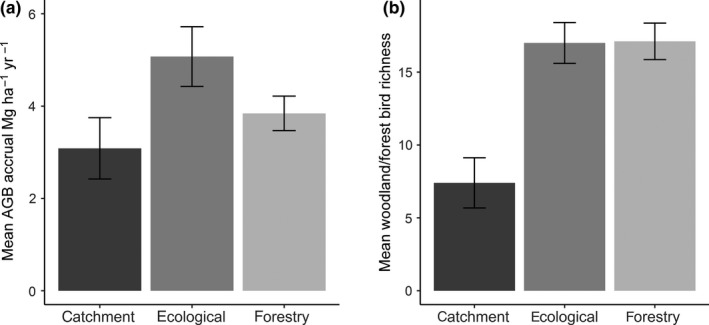
(a) Mean aboveground biomass (AGB) accrual (Mg ha^−1^ year^−1^) and (b) mean forest and woodland‐dependent bird richness for catchment plantings (*n* = 5), ecological plantings (*n* = 16), and forestry plantings (*n* = 9) with standard error bars. No significant difference was detected among planting types for mean AGB accrual (*F* = 2.12, *df* = 27, *p* = .14). Significant difference detected among planting types for mean woodland and forest bird richness (*F* = 8.12, *df* = 27, *p* = .002), with significant pairwise differences between catchment and ecological plantings (*p* < .001), and catchment and forestry plantings (*p* = .001)

A total of 100 tree species (including four exotic) and 53 shrub species (including eight exotic) were recorded across the 30 sites. The woody plant richness ranged from 4 to 30, and aboveground biomass accrual ranged from 0.79 to 8.95 Mg ha^−1^ year^−1^. Mean (±*SE*) aboveground biomass accrual for ecological plantings (5.07 ± 0.65 Mg ha^−1^ year^−1^) was higher than forestry plantings (3.84 ± 0.37 Mg ha^−1^ year^−1^), and lowest for catchment plantings (3.09 ± 0.66 Mg ha^−1^ year^−1^); however, no significant differences were detected among planting types (Figure 5a).

A total of 111 bird species were observed within the sites, including 82 woodland and forest, and 29 open country or waterbird species, and one introduced species. Species in the functional groups included 20 insectivorous, 12 understorey‐nesting, and 59 small‐bodied birds. Some of these species were included in more than one functional group (bird species list available via the Dryad Digital Repository). Mean woodland and forest bird richness for both ecological and forestry plantings (17 ± 1.4 and 17.11 ± 1.25, respectively) were higher than catchment plantings (7.4 ± 1.72). Planting type significantly influenced bird richness (*F* = 8.12, *df* =27, *p* = .002), with pairwise differences detected between ecological and catchment plantings (*p* < .001), and forestry and catchment plantings (*p* = .001; Figure 5b).

### 3.1 Component model of aboveground biomass accrual

**Figure 6:**
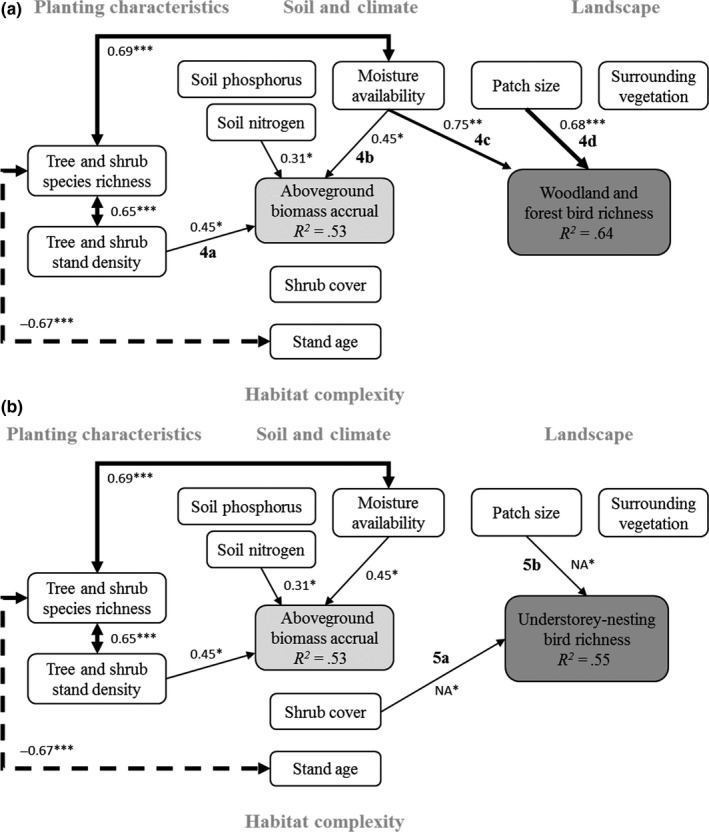
Structural equation modeling results showing significant causal paths between variables and (a) woodland and forest bird richness, and (b) understorey‐nesting bird richness. Black and dashed lines indicate significant positive and negative effects, respectively. Nonsignificant paths are not shown. Given are the standardized regression coefficients and the respective statistical significance (*p < .05, **p < .01, ***p < .001) for each path, and the explained variation (*R* ^2^) for each response variable. Line thickness of each path is proportional to the respective regression coefficient. Letters adjacent to selected paths correspond to the plots shown in Figures 7 and 9, respectively

**Figure 7:**
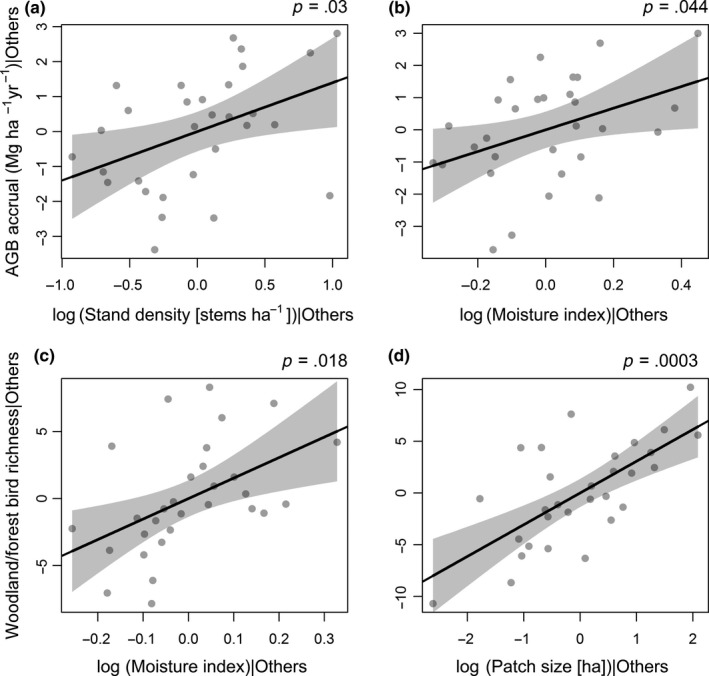
Significant partial relationships of (a) tree and shrub stand density and (b) moisture index on aboveground biomass (AGB) accrual, and (c) moisture index and (d) patch size on woodland and forest bird richness, with fitted regression line and associated 95% confidence intervals from the component model of the woodland and forest bird structural equation model. The plotted variables are the residuals of the original variable given all other independent variables in the component model (partial residuals)

In all four SEMs, the component models predicting aboveground biomass accrual were identical. Aboveground biomass accrual was significantly positively related to stand density (standardized coefficient [*β*_std_] = 0.45, *p* = .03), total N (*β*_std_ = 0.31, *p* = .046), and moisture index (*β*_std_ = 0.45, *p* = .044; Figures 6a and 7a,b). Aboveground biomass accrual increased by approximately 4 Mg ha^−1^ year^−1^ from a moisture index of 0.5 to 1.4 in a log‐linear relationship. Our inclusion of correlation terms among some of the explanatory variable was supported. Tree and shrub richness was strongly and positively correlated with stand density and moisture index (*β*_std_ = 0.65 and 0.69, respectively, both *p* < .001), and negatively associated with stand age (*β*_std_ = −0.67, *p* < .001; Figure 6a). While there was no direct relationship between woody plant richness and aboveground biomass accrual, there was an indirect association via stand density.

### 3.2 Component model of woodland and forest bird richness

We did not find any significant effects of aboveground biomass accrual, shrub cover, patch age or area of surrounding native vegetation on woodland and forest bird richness. Instead, woodland and forest bird richness was strongly positively related to moisture index (*β*_std_ = 0.74, *p* = .018) and patch size (*β*_std_ = 0.69, *p* < .001; Figures 6a and 7c,d). Woodland and forest bird richness increased by approximately 15 species from a moisture index of 0.5 to 1.4 in a log‐linear relationship. d‐sep tests indicated that the SEM fitted the data adequately (Fisher's *C* = 13.13, *df* = 16, *p* = .66).

### 3.3 Component models of understorey‐nesting, insectivorous and small‐bodied bird richness

**Figure 8:**
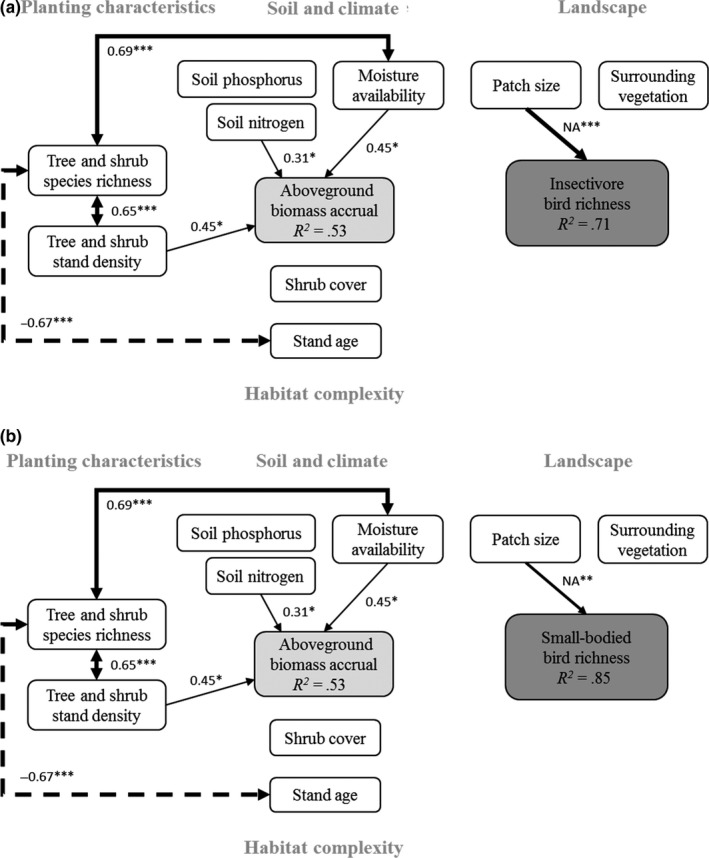
Structural equation modeling results showing significant causal paths between vegetation attributes, soil and landscape variables, and (a) insectivorous bird richness, and (b) small‐bodied bird richness. Black and dashed lines indicate significant positive and negative effects, respectively. Nonsignificant paths are not shown. Given are the standardized regression coefficients and the respective statistical significance (*p < .05, **p < .01, ***p < .001) for each path, and the explained variation (*R* ^2^) for each response variable. Line thickness of each path is proportional to the respective regression coefficient

Insectivorous and small‐bodied bird richness were only significantly associated with patch size (*p* = .001 and *p* = .007, respectively, Figure 8a,b). As indicated by the d‐sep tests, both SEMs fitted the data adequately (insectivorous bird: Fisher's *C* = 12.98, *df* = 16, *p* = .67; small‐bodied bird: Fisher's *C* = 12.08, *df* = 16, *p* = .74).

**Figure 9:**
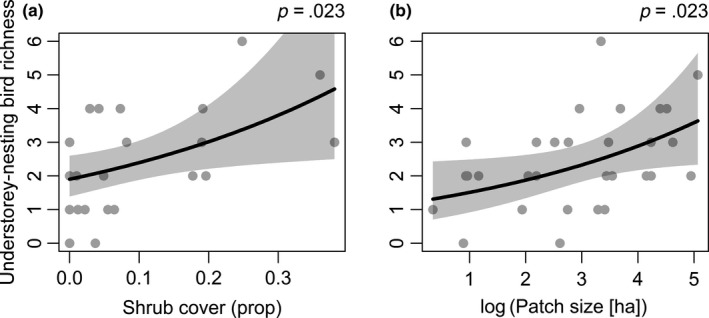
Significant raw relationships of (a) shrub cover and (b) patch size on understorey‐nesting bird richness, with fitted regression line and associated 95% confidence intervals from the component model of understorey‐nesting bird richness

By comparison, for understorey‐nesting bird richness (Figure 6b), shrub cover had a significant positive effect on species richness (Figure 9a, *p* = .023), as did patch size (Figure 9b, *p* = .023). d‐sep tests indicated that the understorey‐nesting bird richness SEM fitted the data as well as the other SEMs (Fisher's *C* = 13.68, *df* = 16, *p* = .62). Moisture index was not a clear predictor of understorey‐nesting, insectivorous or small‐bodied bird richness.

## 4 DISCUSSION

### 4.1 Drivers of aboveground biomass accrual

Our results show that water availability limits aboveground biomass accrual in restoration plantings in subtropical Australia. This has also been shown in studies of aboveground biomass annual increment or carbon stocks in subtropical and tropical forest biomes that have similar rainfalls (MAP > 800 mm and >1,300 mm, respectively) to our study sites (MAP 735–1,660 mm; Chen et al., 2018; Sullivan et al., 2017). Consistent with an Australian‐wide study of native mixed‐species plantings in Australia (Paul, Cunningham, et al., 2016), aboveground biomass accrual in our study increased with stand density; however, growth is density dependent to some extent, and this relationship is likely to weaken as plantings mature. In fact, thinning can increase the growth rates of retained trees and has been found to accelerate productivity in dense natural regeneration of Brigalow forest in subtropical Australia (Dwyer, Fensham, & Buckley, 2010).

We did not detect a direct positive plant diversity–productivity relationship, as reported after eight years in a subtropical forest planting experiment in China (Huang et al., 2018). However, overall ecological plantings (ranging from 4 to 16 years, and characterized by higher tree and shrub richness and stand density), had higher aboveground biomass accrual than forestry or catchment plantings (ranging from 14 to 25 years; Figure 5a). Other studies have failed to find a relationship between woody plant diversity and aboveground biomass accumulation in plantings across a wide climate gradient in Australia, at least in the first few decades of growth (Paul, Cunningham, et al., 2016; Staples, Dwyer, England, & Mayfield, 2019). In natural forests, diversity effects on productivity tend to increase as communities mature (Duffy et al., 2017; Meyer et al., 2016; Reich et al., 2012). Strong abiotic forces, including climate and nutrient availability, can also mask biodiversity effects (Duffy et al., 2017). Because woody plant richness and aboveground biomass accrual in our study were both strongly affected by moisture availability, this likely weakened the richness–productivity relationship. While plant richness was not a direct predictor of productivity, it was indirectly related via a significant, positive association with stand density.

We found soil N to have a significant positive effect on aboveground biomass accrual. There was no significant effect of extractable P on aboveground biomass accrual; however, Australia has a high proportion of soils that have formed on sedimentary rocks with low P content, or highly weathered soils where much P has been lost through leaching (Handreck, 1997). As a response, many native tree species have adapted to low P availability through effective acquisition strategies, and tree growth can be buffered from P limitation (Cheesman et al., 2018).

### 4.2 Drivers of bird richness

**Figure 10:**
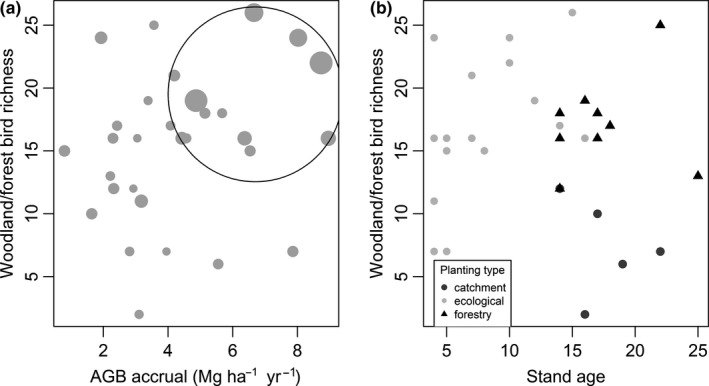
Woodland and forest bird richness plotted with (a) aboveground biomass (AGB) accrual across all restoration sites (point size corresponds to moisture index and cluster of wetter sites with both high AGB accrual and woodland and forest bird richness are circled), and (b) patch age across the three different restoration types (catchment, ecological, and forestry plantings)

Our results suggest that water availability influences bird diversity directly, rather than indirectly via plant productivity, such that wetter areas support more species, consistent with findings by Hawkins et al. (2005) across Australia. A more recent study found that bird richness in Australia was positively related to evapotranspiration at the continental scale, and vegetation productivity and structure at regional scales, suggesting that species richness is influenced by water availability either directly or indirectly, depending on the scale (Coops et al., 2018). Therefore, it may be that climate drivers are masking vegetation effects on bird richness at the scale of our study. However, it is possible to achieve high values of both aboveground biomass accrual, and woodland and forest bird richness at wetter sites (Figure 10a), indicating synergies between aboveground biomass accrual and bird richness at sites where water is not limiting. Furthermore, shrub cover was found to be an important variable influencing understorey‐nesting bird richness, expected due to the reliance of this group on understorey vegetation for nesting and foraging (Martin & McIntyre, 2007). In terms of managing plantings, livestock grazing is likely to remove shrubs and therefore reduce understorey‐nesting bird richness. The removal of shrubs may also decrease small‐bodied bird richness from competitive exclusion by aggressive noisy miners (Martin & McIntyre, 2007; Val, Eldridge, Travers, & Oliver, 2018).

We found that larger patches had greater species richness for all bird groups examined, consistent with island‐biogeography theory (MacArthur & Wilson, 1967) and species–area relationships (Rosenzweig, 1995). While it is possible we encountered more bird species from covering more area in larger patches, our results likely represent the species present across the patch (and not within a quadrat), which is useful for management of the site, such as planning understorey restoration. Furthermore, patch size has been found to predict bird richness in a study of forest plantings in fragmented landscapes of southeastern Australia, employing both fixed‐time and fixed‐area bird surveys (Kavanagh et al., 2007; Lindenmayer et al., 2010).

The area of native vegetation in the landscape was not significantly related to any of our bird richness variables. This is surprising given that the amount of surrounding native vegetation had a strong influence on woodland‐dependent bird richness in natural regeneration of subtropical Brigalow and eucalypt forests elsewhere in southern Queensland (Bowen et al., 2009). Some of our sites were also wet forest plantings, however, surrounding vegetation cover has also been found to predict forest‐dependent bird richness in tropical rainforest plantings in Australia and Costa Rica (Freeman et al., 2015; Reid, Mendenhall, Rosales, Zahawi, & Holl, 2014). Many of our sites contained native vegetation that was not mapped as remnant or mature regrowth (i.e., native plantation, young regrowth, recent planting), which may have underestimated the area of native vegetation in the spatial analysis. Alternatively, other landscape context factors, such as distance to water, may be more important for bird richness in subtropical climates, as found in temperate woodland plantings in Australia (Lindenmayer et al., 2010).

Despite patch age influencing bird diversity within forestry plantings in subtropical Australia (Law, Chidel, Brassil, Turner, & Kathuria, 2014), patch age had no effect on bird richness variables in our study. This is likely because our study sites contained a mixture of planting types, and many of our older sites were less diverse forestry plantings with open understoreys, which supported similar woodland and forest bird richness as younger, more diverse ecological plantings (Figures 5b and 10b). Likewise to our findings, a study in a cleared agricultural region in southeast Australia, also found that ecological plantings between 4–8 years contained a similar bird composition to forestry plantings of 11–15 years (Munro et al., 2011), indicating that colonization depends on the quality of restored habitat (Lindenmayer, Blanchard, et al., 2018). This suggests it is possible to achieve reasonable bird diversity in ecological plantings within 4–16 years by planting a mixture of native trees and shrubs. Further monitoring of bird communities in these younger, ecological plantings would be desirable to detect changes as they mature. Indeed, some bird species can rapidly colonize restored forests and woodlands. For example in Australia, small‐bodied species recolonized restored temperate woodland understories within 6–8 years (Lindenmayer, Blanchard, et al., 2018), and half of the rainforest bird species known from adjacent old‐growth habitat recolonized rainforest plantings within 10 years (Freeman et al., 2015). Elsewhere, woodland generalist species recolonized natural woodland regeneration within 10 years in the United Kingdom (Whytock et al., 2018). However, the age range of our plantings was relatively small (4–25 years). It can take many decades for a forest to mature, and colonization by specialist bird species is likely to take much more than 20 years (Catterall et al., 2012; Freeman et al., 2015). Recent results from a chronosequence (20–120 years) of secondary tropical forests in Panana suggest that connectivity with extensive primary forest is a more important determinant of bird species richness than forest age (Mayhew, Tobias, Bunnefeld, & Dent, 2019), therefore the effect of landscape context in our study warrants further investigation.

## 5 CONCLUSIONS

Our results suggest that planting large patches in regions of high water availability would increase both aboveground biomass accrual and bird richness in subtropical Australia, thus providing synergies for carbon and biodiversity. Planting at high densities is also likely to enhance aboveground biomass accrual, and inclusion of a shrub layer to establish structural diversity would be required to support colonization by understorey‐nesting birds. To accelerate the recovery of bird diversity, we recommend diverse plantings of native trees and shrubs, which yield comparable rates of aboveground biomass accrual to more production‐focused plantings.

## CONFLICT OF INTEREST

None declared.

## AUTHOR CONTRIBUTIONS

VH, KW, and JMD conceived the ideas, VH, JMD, and JRE designed the methodology; VH collected and analyzed the data; JMD and JRE contributed analysis techniques; VH wrote the manuscript; JMD, JRE and KW contributed critically to the drafts.

## Data Availability

Biomass estimates, bird survey data, and SEM variable data are available via Dryad Digital Repository, https://doi.org/10.5061/dryad.98sf7m0dw.
